# Telomere status in chronic lymphocytic leukemia with *TP53* disruption

**DOI:** 10.18632/oncotarget.10927

**Published:** 2016-07-29

**Authors:** Romain Guièze, Mélanie Pages, Lauren Véronèse, Patricia Combes, Richard Lemal, Mathilde Gay-bellile, Martine Chauvet, Mary Callanan, Fabrice Kwiatkowski, Bruno Pereira, Philippe Vago, Jacques-Olivier Bay, Olivier Tournilhac, Andreï Tchirkov

**Affiliations:** ^1^ CHU Clermont-Ferrand, Hématologie Clinique, Clermont-Ferrand, France; ^2^ EA 7283 CREaT, Université d’Auvergne, Clermont-Ferrand, France; ^3^ Department de Neuropathologie, Hôpital Sainte-Anne, Paris, France; ^4^ Université Paris Descartes, Paris, France; ^5^ Université Clermont 1, UFR Médecine, Cytologie Histologie Embryologie Cytogénétique, Clermont-Ferrand, France; ^6^ CHU Clermont-Ferrand, Cytogénétique Médicale, Clermont-Ferrand, France; ^7^ EA 4677 ERTICa, Université d’Auvergne, Clermont-Ferrand, France; ^8^ Inserm U823, Institut Albert Bonniot & Université Joseph Fourier, Grenoble, France; ^9^ CHU Grenoble, Laboratoire de Génétique Onco-hématologique, Grenoble, France; ^10^ Centre Jean Perrin, Clermont-Ferrand, France; ^11^ Direction de la Recherche Clinique et de l’Innovation, Département de Biostatistiques, CHU Clermont-Ferrand, Clermont Ferrand, France

**Keywords:** chronic lymphocytic leukemia, *TP53*, telomere, *hTERT*, shelterin

## Abstract

In chronic lymphocytic leukemia (CLL), telomere dysfunction is associated with poor outcomes. *TP53* is involved in cellular responses to dysfunctional telomeres, and its inactivation is the strongest adverse prognostic factor for CLL. Given the biological relationship between *TP53* and telomeres, and their prognostic value, it is important to improve our understanding of the impact of *TP53* alterations on telomeres. We performed a comprehensive study of the deletions and mutations of the *TP53* gene and telomere parameters, including *hTERT* and the shelterin complex, in 115 CLL patients. We found that any type of *TP53* alteration was associated with very short telomeres and high *hTERT* expression, independently of other biological CLL features. Patients with disrupted *TP53* showed telomere deletions and chromosomal end-to-end fusions in cells with complex karyotypes. *TP53* disruption was characterized by downregulation of shelterin genes. Interestingly, low expression of *POT1*, *TPP1* and *TIN2* was also found in some patients with wild-type *TP53* and had an adverse impact on progression-free survival after standard genotoxic therapy. In conclusion, we have demonstrated that patients with disrupted *TP53* have severe telomere dysfunction and high genomic instability. Thus, the telomeric profile could be tested as a biomarker in CLL patients treated with new therapeutic agents.

## INTRODUCTION

Telomeres protect chromosomal ends and are comprised of tracts of G-rich nucleotide repeats bound by a protein complex, shelterin, containing TRF1 and TRF2 (telomeric repeat binding factors 1 and 2), POT1 (protection of telomeres 1), TIN2 (TRF1-interacting protein 2), TPP1 (POT1–TIN2 organizing protein, also known as ACD) and RAP1 (repressor-activator protein 1) [1, 2].

The protective function of telomeres requires a sufficient amount of telomeric repeats and integrity of the shelterin complex. Telomere shortening due to incomplete replication of telomeric DNA, as well as shelterin defects, result in telomere dysfunction, which can lead to chromosomal and genomic instability. However, cells with dysfunctional telomeres are removed via senescence or apoptosis, and the p53 protein, a guardian of genomic integrity, regulates this physiological response. *hTERT* (human telomerase reverse transcriptase) is a key component of telomerase, which can regenerate and stabilize shortened telomeres, enabling unlimited cell proliferation. Telomere dysfunction, p53 deficiency and *hTERT* activation cooperate in oncogenesis, promoting genetically unstable and immortal tumor cell clones [3–5].

In chronic lymphocytic leukemia (CLL), telomere shortening and *hTERT* expression are generally observed in patients with advanced disease and/or high-risk biological features, such as unmutated immunoglobulin heavy chain variable (IGHV) genes, but some early-stage patients can also display short telomeres and high *hTERT*. Telomere shortening and *hTERT* expression have been also related to poor outcomes [6–10]. The shelterin complex is globally dysregulated at the transcriptional level [11, 12] and the presence of dysfunctional telomeres has been correlated with a downregulation of shelterin genes [11, 13], but the relationship between shelterin status and the clinico-biological parameters and outcomes of CLL remain unknown.

Previous reports suggest that CLL patients with *TP53* loss, due to del(17p), may show excessive telomere shortening compared to other cytogenetic subgroups [12, 14–16]. Deletion of 17p and also *TP53* mutations in the absence of del(17p) are found in 10–15% of untreated CLL patients, and represent the strongest adverse prognostic factor to predict early resistance to standard genotoxic agents and a poor outcome [17, 18]. Given the high prognostic significance of *TP53* and telomere status and their cooperation in oncogenesis, we investigated the potential link between *TP53* gene alterations and impaired telomeres in CLL. We performed a comprehensive study of deletions and mutations of the *TP53* gene and of telomere parameters, including assessment of telomeres as well as *hTERT* and shelterin gene expression in 115 CLL patients.

## RESULTS

### TP53 mutations and deletions are associated with short telomeres and hTERT overexpression

**Figure 1:**
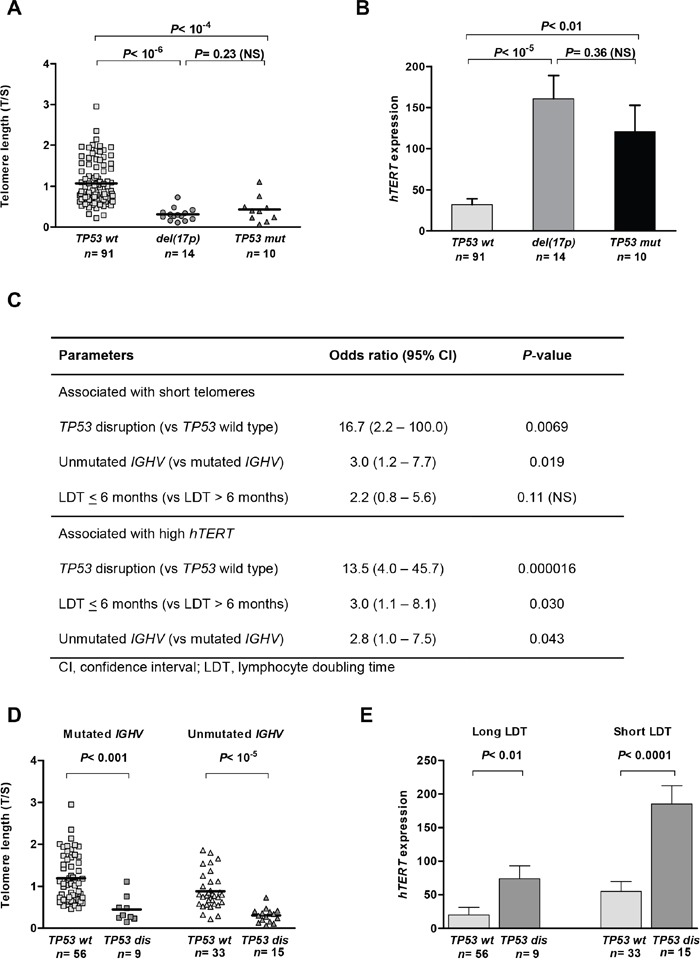
*TP53* loss and mutation are equally associated with telomere shortening and *hTERT* overexpression in chronic lymphocytic leukaemia (CLL) Mean telomere length **A.** and *hTERT* expression **B.** in patients with wild-type (wt) *TP53* and in patients with either del(17p) or *TP53* mutations (mut) in the absence of del(17p) (Kruskal–Wallis H test). The impact of *TP53* disruption (dis) was independent of *IGHV* status and lymphocyte doubling time (LDT), as shown in the multiple logistic regression, with an association between shorter (<0.925, mean) telomeres and higher (>55.5, mean) *hTERT* **C.** This is illustrated by comparing telomere lengths in patients with mutated and unmutated *IGHV* **D.** and *hTERT* expression to patients with long and short LDTs **E.** Bars correspond to the standard error of the mean.

*TP53* mutations were found in 20 patients (17.4%): of these, 10 also had a del(17p). Four additional cases had a del(17p) only. We found that telomere length and *hTERT* expression did not differ between patients with a del(17p) and those carrying a *TP53* mutation in the absence of a 17p deletion (Figure 1A-1B). In contrast, telomere length in these cases was considerably shorter and *hTERT* expression was higher compared to that in patients with wild-type (wt) *TP53*. In further analyses, we considered *TP53* mutated and/or deleted cases as a single *TP53*-disrupted subgroup (*n*=24).

We then investigated the impact of *TP53* status on telomeres in relation to other adverse biological features of CLL, which are known to correlate with telomere length and *hTERT* expression [14, 19]. In our cohort, short telomere length was, as expected, associated with unmutated *IGHV* genes (*P*= 0.00096) and short lymphocyte doubling time (LDT; *P*= 0.00083). *hTERT* overexpression was found in patients with unmutated *IGHV* (*P*= 0.0032) and short LDTs (*P*= 0.0016). Although all these characteristics increased the risk of having short telomere length and high *hTERT* expression, *TP53* disruption had the strongest impact in the multiple logistic regression analyses (Figure 1C). This is illustrated in Figure 1D, showing that telomere length was significantly shorter in patients with a *TP53*-disrupted status compared to a *TP53* wild-type status, irrespective of the *IGHV* mutational status. Similarly, *hTERT* expression was significantly higher in patients with *TP53* disruption compared to those with a wild-type *TP53* status in the subgroups that had either a long or a short LDT (Figure 1E).

We also compared telomere lengths and *hTERT*-expression levels between cytogenetic CLL subgroups. There was gradual and significant telomere shortening, and an increase in *hTERT* from low-risk subgroups to a *TP53*-disrupted subgroup (Supplementary Figure S1A-S1B). In addition, the mean number of karyotype aberrations increased gradually and was greatest in patients with disrupted *TP53* (Supplementary Figure S1C).

### Telomere abnormalities in metaphase from TP53-disrupted cases

**Figure 2:**
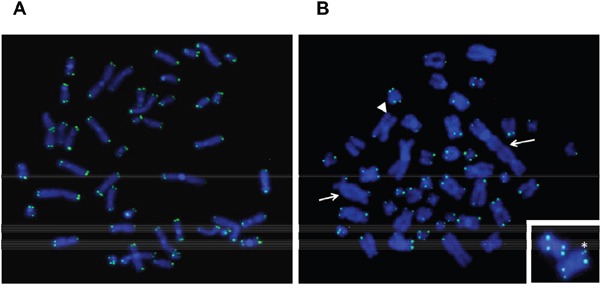
Pantelomeric FISH of representative metaphases from wild-type (wt) *TP53* **A.** and disrupted *TP53* **B.** *TP53* disruption was associated with telomeric fusions that led to dicentric chromosomes (arrows) and frequent loss of telomeric signals (one of them is shown with an arrowhead). A duplication of a telomeric signal is shown with an asterisk.

We performed pantelomeric FISH analysis on six *TP53*-disrupted cases and six *TP53* wild-type cases (Figure 2). The mean number of telomere signal losses, representative of severe telomere erosion (Figure 2B), was significantly higher in *TP53*-disrupted than in wild-type *TP53* cases (5.6 vs 1.1, respectively, *t*-test, *P* <0.01). In contrast, no difference was observed in the frequency of multiple telomeric signals, which are markers of telomere replication defects: 0.7 in *TP53*-disrupted cases vs 0.65 in wild-type *TP53* cases, NS. In addition, nine out of 24 *TP53*-disrupted cases had complex karyotypes, and four of these showed telomeric fusions (Figure 2B), which are characteristic of extreme telomere erosion, thus highlighting the association between telomeric and chromosomal instability.

### Low shelterin gene expression in patients with TP53 disruption

**Figure 3:**
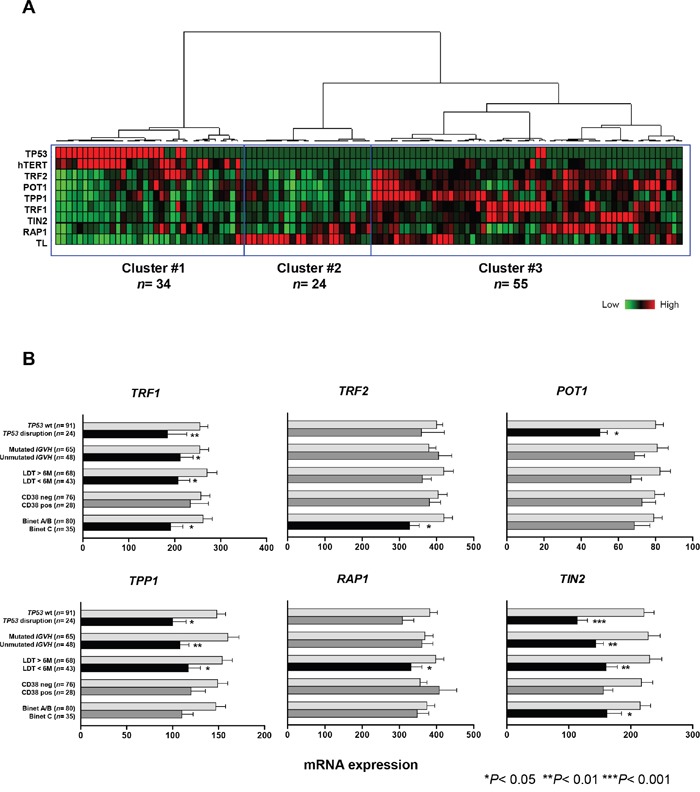
Low shelterin-gene expression in CLL patients with disrupted *TP53* and in patients with adverse prognostic factors **A.** Hierarchical clustering of 115 CLL patients according to *TP53* status, telomere length and expression levels of *hTERT* and shelterin genes (*TRF1*, *TRF2*, *POT1*, *TPP1*, *RAP1*, *TIN2*). Patients with disrupted *TP53* (red) are clustered into a distinct group with short telomeres, high *hTERT* and low levels of shelterin genes. **B.** Mean expression levels of shelterin genes in different CLL subgroups according to *TP53* status, *IGHV* mutation profile, CD38 expression, lymphocyte doubling time (LDT) and Binet stage (H test). Bars correspond to the standard error of the mean.

We first performed unsupervised hierarchical clustering analysis that included *TP53* status and telomere characteristics: i.e. shelterin and *hTERT* gene-expression levels and relative telomere length (Figure 3A). This approach identified three distinct groups. Cluster #1 (*n*= 34) was characterized by shortened telomeres, high *hTERT* and low shelterin gene expression. This cluster included 22 of the 24 patients with *TP53* disruption. Interestingly, we also observed a smaller group of patients with wild-type *TP53* and without *hTERT* overexpression and telomere shortening, but with downregulated shelterin genes (cluster #2, *n*= 24). Finally, there was a large group (cluster #3, *n*= 55) that had heterogeneous telomere lengths, low *hTERT* and relatively high levels of shelterin gene expression. Overall, *TP53* disruption was clearly associated with low shelterin gene expression, but a similar downregulation was also found in a proportion of wild-type *TP53* cases.

The expression of shelterin genes has not yet been correlated with established CLL prognostic factors; we performed this analysis in our series (Figure 3B). Significant downregulation of several shelterin genes were found in patients in Binet C stage (*TRF1*, *TRF2* and *TIN2*). A short LDT was associated with significant downregulation of all the shelterin genes except for *TRF2* and *POT1*. Expression of *TRF1*, *TPP1* and *TIN2* was lower in cases with unmutated *IGHV*. *TP53*-disruption was also significantly associated with lower expression of *TRF1*, *POT1*, *TPP1* and *TIN2*. This was independent of the type of *TP53* alteration (data not shown).

Regarding the correlation with cytogenetic subgroups, the changes to shelterin gene expression were not consistent, except from *TIN2*, which showed highly significant gradual reduction from a normal/del(13q) pattern to a *TP53*-disruption subgroup, in parallel with telomere shortening (Supplementary Figure S2).

### Prognostic value of telomere length and shelterin gene expression in treated patients with wild-type *TP53*

**Figure 4:**
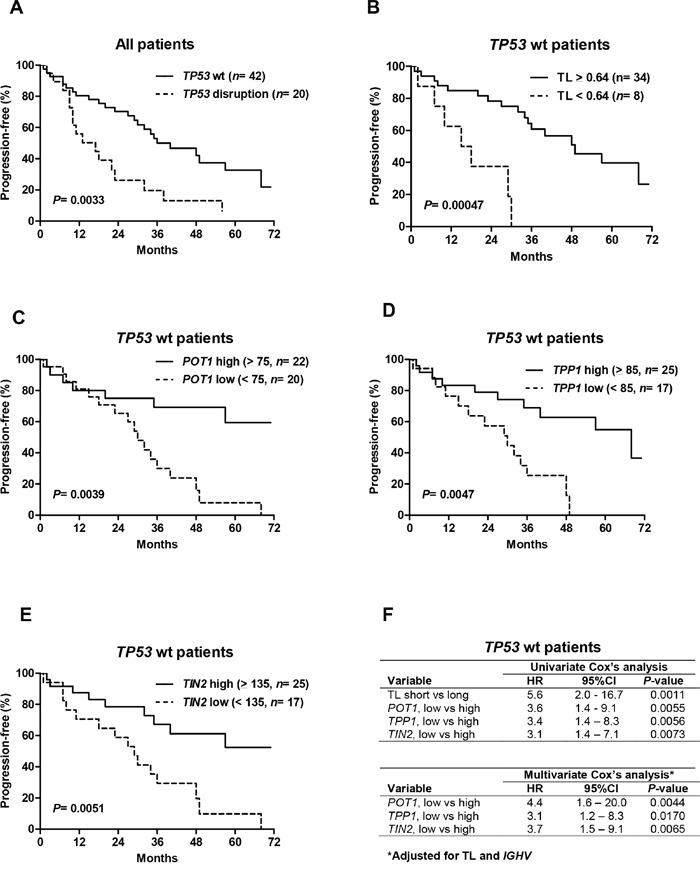
Telomere length and shelterin expression predict progression-free survival (PFS) in patients with wild-type (wt) *TP53* **A.** Kaplan–Meier estimates of PFS in the whole patient population as a function of *TP53* status and the wild-type *TP53* cohort according to telomere length **B.** *POT1* **C.** *TPP1* **D.** and *TIN2* **E.** levels. The optimal cut-off values were determined using recursive partitioning. CLL patients with short telomeres and low *POT1, TPP1* and *TIN2* levels had significantly shorter PFSs. **F.** Corresponding hazard ratios (HR) and 95%-confidence intervals (95%CI) are presented. When adjusted for telomere length and *IGHV* mutation profile, low *POT1*, *TPP1* and *TIN2* expression remained significant predictors for a shorter PFS.

As expected, survival analyses in treated patients confirmed the significantly shorter progression-free survival (PFS) of patients with disrupted *TP53* compared to patients with wild-type *TP53* (*P*= 0.0033; Figure 4A). In addition, telomere length, *hTERT* and three shelterin-gene (*POT1*, *TPP1*, *TIN2*) transcript levels were also significant predictors of PFS (data not shown), but these parameters were strongly correlated with *TP53* status. We therefore investigated whether these telomere characteristics had an additional prognostic value beyond classifying patients into *TP53*-disrupted or *TP53*-wild-type subgroups. We performed survival analysis among patients with wild-type *TP53* and found that short telomeres (*P*= 0.00047, Figure 4B), low *POT1* (*P*= 0.0039; Figure 4C), low *TPP1* (*P*= 0.0047; Figure 4D) and low *TIN2* (*P*= 0.0051; Figure 4E) identified subsets of patients with significantly shorter survival times. These parameters were also associated with a higher risk of progression in the univariate Cox's analysis (Figure 4F). A multivariate Cox's analysis of known poor prognostic CLL features (*IGHV* status, LDT, CD38, Binet stage) showed that unmutated *IGVH* genes significantly and independently predicted PFS in this patient subgroup with the hazard ratio of 2.2 (95% CI: 1.0 to 4.3, P= 0.022). When adjusted for *IGHV* mutation status and telomere length, shelterin transcript levels remained significant predictors of PFS in patients with wild-type *TP53* (Figure 4F).

## DISCUSSION

We have shown that CLL patients with disrupted *TP53* have severe telomere shortening associated with *hTERT* overexpression and downregulation of shelterin genes. This telomere profile was found in patients with *TP53* mutations in the absence of del(17p) and in patients with concomitant *TP53* mutations and del(17p) or del(17p) alone: this suggests that any alteration to *TP53* leads to important telomere dysfunction in CLL.

Both *TP53* alteration and short telomeres have been previously correlated with progressive CLL, and with unfavorable biological factors such as unmutated *IGHV* and a high proliferation rate [14, 19, 20]. We have demonstrated that the association of *TP53* disruption with short telomeres and high *hTERT* was an independent factor in this adverse biological profile, and that *TP53* was the strongest determinant of telomere status and *hTERT* expression. We have also shown that patients with disrupted *TP53* had the shortest telomeres among the cytogenetic subgroups; this association has been previously described for a 17p deletion [16], but has not yet been reported for mutated *TP53*. Patients with disrupted *TP53* also had the highest levels of *hTERT* among the subgroups.

Using pantelomeric FISH, we found that *TP53* disruption was associated with increased numbers of telomeric deletions, and we also observed chromosomal end-to-end fusions in patients with disrupted *TP53* and complex karyotypes. These findings illustrate that impaired p53 pathways can allow continuous cell division despite telomere shortening, and the accumulation of cells with critically eroded telomeres, thus leading to chromosomal fusions and genomic instability. In addition, wild-type p53 is a powerful inhibitor of *hTERT*, and loss of this function can also enhance expression of *hTERT*, which is essential for survival and replication of tumor cells [21]. Telomere attrition and *TP53* disruption may thus represent two important cooperating events of tumor progression in CLL, as has been previously shown in epithelial tumor models [4, 22].

Telomere shortening in CLL patients with disrupted *TP53* was also associated with low expression of *TRF1*, *POT1*, *TPP1* and *TIN2* shelterin genes. Using clustering analysis, we found that almost all cases of disrupted *TP53* were within a distinct subset that had short telomeres and downregulated shelterin genes. The abnormal expression of shelterin genes, in the context of p53-deficiency and short telomeres, may have contributed to the higher levels of genomic instability in this CLL subset.

Clustering analyses indicated that some patients with wild-type *TP53* also showed shelterin downregulation, but this low expression was independent of telomere shortening. Moreover, we also found significantly lower shelterin gene expression in cases with adverse clinical and biological parameters, such as advanced Binet stage, unmutated *IGHV* and short LDTs. Interestingly, shelterin status was not consistently correlated with the cytogenetic subgroups, with the exception of *TIN2*. These results highlight the heterogeneity of shelterin status in CLL and suggest the complexity that underlies these mechanisms.

The factors regulating shelterin genes remain unknown. Nevertheless, some events that could affect their function and expression in cancer, such as mutation and DNA methylation, have been described. Somatic mutations of *POT1* have been reported in CLL, and this represents the first description of a shelterin complex-gene mutation in human cancer [23]. CLL cells with *POT1* mutations display multiple telomeric and chromosomal aberrations, suggesting that these mutations and the subsequent alterations to shelterin can favor the acquisition of the malignant features seen in CLL cells.

In breast-cancer cell lines, a significant reduction in *POT1* mRNA level was associated with *POT1* promotor methylation [24]. Interestingly, in the clonal evolution of CLL, a *POT1* mutation seems to precede the appearance of alterations to *TP53* [26]. In addition, downregulation of shelterin genes can occur prior to telomere shortening in early CLL and so contribute to the reduction in telomere protection [13]. Sequential analyses of patients with low levels of gene expression of shelterin would be of interest to investigate the evolution of telomere status and the timing of *TP53* appearance and other alterations, as well as the onset of chromosomal instability.

Finally, we found a significant adverse impact of short telomeres and low *POT1*, *TPP1* and *TIN2* on PFS of the CLL patients in our cohort, who had been treated mainly with standard genotoxic agents. This impact was found in the whole population and also in patients with wild-type *TP53*, emphasizing the additional prognostic value of these parameters. In particular, the significant impact of shelterin gene expression on survival in CLL has not been described previously. Of note, these shelterin components regulate telomerase recruitment via POT1, and TIN2 recruits the TPP1–POT1 complex, constituting a bridge between the different shelterin components [2]. Unlike *POT1*, mutated *TIN2* and *TPP1* have not been observed in tumors, but germline mutations of these genes have been reported in human genetic diseases leading to bone-marrow failure [26, 27]. Taken together, these observations suggest that *POT1*, *TIN2* and *TPP1* could be key-components in the shelterin complex implicated in telomere dysfunction and adverse outcomes in CLL.

Further studies of patients with downregulated shelterin genes are warranted for mechanistic understanding of the role of telomere abnormalities in CLL. In addition to *TP53* disruption, these investigations have to take into account other recurrent CLL mutations with an emphasis on telomeres, such as altered *ATM* and *SF3B1* genes and, as stated above, the *POT1* gene. Mutations in the *ATM* and *SF3B1* genes are known to be associated with shorter telomeres in CLL cells [28, 16]. ATM, a major DNA damage sensor, appears to play a complex role in telomere protection and length regulation trough interactions with the shelterin complex [29]. A possible telomeric role of the SF3B1 spliceosome protein was recently revealed by the transcriptomic study of *SF3B1*-mutated CLL cells, showing an overexpression of the *TERC* gene encoding telomerase RNA template, which could lead to aberrant telomerase regulation [30].

In conclusion, we have demonstrated that CLL patients with disrupted *TP53* show severe dysfunction of telomeres, involving telomeric DNA, *hTERT* and shelterin. This telomeric dysfunction can contribute to enhanced genomic instability and, consequently, to treatment resistance in CLL. Further studies are required to evaluate the impact of *TP53* in the context of other molecular alterations, in particular ‘multiple hit’ events described recently in resistant CLL [31]. Finally, this telomeric profile could be tested as a predictive marker in CLL patients treated with new non-genotoxic therapies.

## MATERIALS AND METHODS

### Patients and samples

After informed consent according to the Declaration of Helsinki, peripheral blood samples were collected from 115 CLL patients at the Clermont-Ferrand University Hospital (France) between 2007 and 2012. The patients' characteristics at the time of sampling are shown in Supplementary Table S1. Diagnosis and treatment requirements were based on the guidelines of the International Workshop on Chronic Lymphocytic Leukemia [32]. A total of 62 patients required treatment, consisting of immunochemotherapy (fludarabine, cyclophosphamide, rituximab *n*= 44, other regimens *n*= 6), alemtuzumab (*n*= 9) or alkylating agent-based regimens (*n*= 3). Median follow-up time from the start of treatment was 41 months. Peripheral blood samples were obtained before treatment. Peripheral blood lymphocytes were isolated by Lymphoprep™. The proportion of tumor cells obtained was >85%.

### Cytogenetic analyses

Analyses were performed at the time of sampling. Karyotype was performed after immuno-stimulation of cell cultures with CpG-oligonucleotide and interleukin 2. Chromosome preparation and staining were done according to standard protocols. Twenty metaphases were analyzed. Complex karyotype was defined as ≥3 abnormalities. Locus-specific fluorescence *in situ* hybridization (FISH) was performed on interphase nuclei and/or metaphases, following standard procedures and using specific probes (co-hybridized with a control probe) to detect deletions of 13q14.3, 11q22.3 and 17p13 (LSI D13S319, LSI ATM and LSI TP53). Trisomy 12 was studied using the centromeric probe D12Z3. All probes were from Vysis (Abbott Molecular, Rungis, France) and used in accordance with the manufacturer's recommended procedures. At least 200 interphase nuclei were examined. The cut-off levels for the presence of aberrations were 7% of positive cells for deletions and 5% for trisomy 12. Patients were classified according to previously reported hierarchical patterns [31]. Pantelomeric FISH was performed with a Telomere PNA FISH Kit (DAKO, Glostrup, Denmark), used in accordance with the manufacturer's recommended procedures. Results were recorded using an Axioplan2 imaging fluorescence microscope (ZEISS, Göttingen, Germany) fitted with appropriate filters, a CCD camera, and the digital-imaging software, Isis, v3.8.8 (Metasystems, Althussheim, Germany).

### *TP53* mutational analyses

Genomic DNA was extracted using a Nucleospin Blood L (Macherey-Nagel) kit. Mutation analyses of *TP53* exons 2–11 were done by DNA direct sequencing using a 3130XL sequencer (Applied Biosystems, Villebon-sur-Yvette, France) and analyzed with Seqscape software. The genomic reference sequence used was IARC NC_000017 v9. The primers were designed to cover all coding exons and intron–exon boundaries. The characteristics and distributions of mutations were assessed using the IARC *TP53* Mutation Database and the Ensembl Database. *TP53* gene mutations found in our CLL cohort are detailed in Supplementary Table S2. Most were deleterious missense mutations located in the DNA-binding domain and have been previously observed in other cancer types.

### Assessment of telomere length using quantitative PCR

Average telomere length was evaluated with quantitative real-time DNA-PCR in a LightCycler 480 System (Roche Diagnostics, Meylan, France) using SYBR Green I technology (SYBR Green Kit, Roche Diagnostics), as described elsewhere [15]. This method measures the relative telomere length as the T/S ratio between the template amounts of telomere repeat (T) and a reference single-copy gene (S) [33]. We used *GAPDH* as a reference gene. Using this approach, we have previously shown that the T/S ratio in tumor cells was proportional to average telomere length as assessed by classical telomere restriction-fragment analyses [15].

### Quantitative RT-PCR for *hTERT* and shelterin complex gene expression

Total RNA was extracted using TRIzol (Fisher Scientific, Illkirch, France) according to standard procedures. Total RNA was converted to cDNA by reverse transcription using Superscript II reverse transcriptase (Invitrogen, Cergy-pontoise, France), according to the manufacturer's instructions. The expression of *hTERT* and the shelterin complex genes *TRF1*, *TRF2*, *POT1*, *TPP1*, *RAP1* and *TIN2* were quantified using real-time RT-PCR in a LightCycler 480 System (Roche Diagnostics, Meylan, France), as described previously [11, 15]. The normalized copy numbers were expressed as the ratio between the numbers of transcript copies of the target and control (*GUSB*) genes, multiplied by 100.

### Statistical analyses

Comparisons of quantitative variables were performed using standard tests. As is usual in exploratory studies, we chose not to adjust probabilities by the Bonferroni method. While decreasing the rate of false positives, this method also increases in a similar proportion the rate of false negatives. Unsupervised hierarchical clustering analysis was performed to identify subclasses with distinct expression of shelterin genes and telomeric-length profiles. Progression-free survival (PFS) was calculated from the date of starting treatment until a relapse, progression or death from any cause. Survival curves were established using the Kaplan–Meier method and compared with the log-rank test. The univariate and multivariate analyses was performed according Cox's regression model. The data were analyzed using SEM software [34].

## SUPPLEMENTARY MATERIALS FIGURES AND TABLES


