# Supplementary material for: Telomere status in chronic lymphocytic leukemia with TP53 disruption
Source: Oncotarget. 2016 Jul 29;7(35):56976–85. doi: 10.18632/oncotarget.10927 (PMC5302966; doi:10.18632/oncotarget.10927)
Supplement: Supplementary file 1 [file oncotarget-07-56976-s001.pdf]

Telomere status in chronic lymphocytic leukemia with *TP53* disruption

SUPPLEMENTARY FIGURES AND TABLES

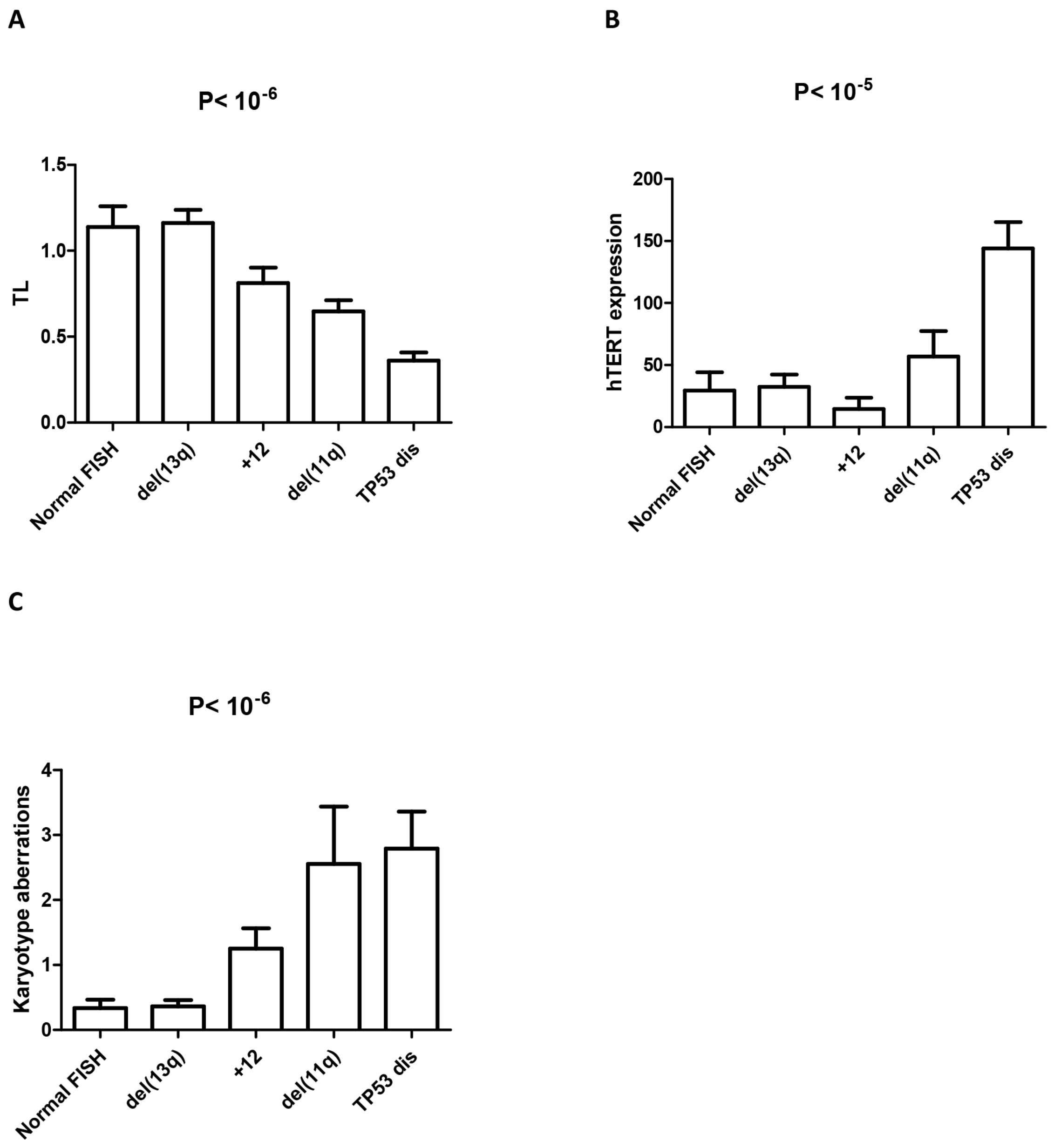

Supplementary Figure S1: *TP53*-disrupted patients showed the shortest telomeres A. the highest *hTERT* expression **B.** and the highest mean number of karyotype aberrations among CLL subgroups **C.** The comparisons were made using the Kruskal-Wallis H test.

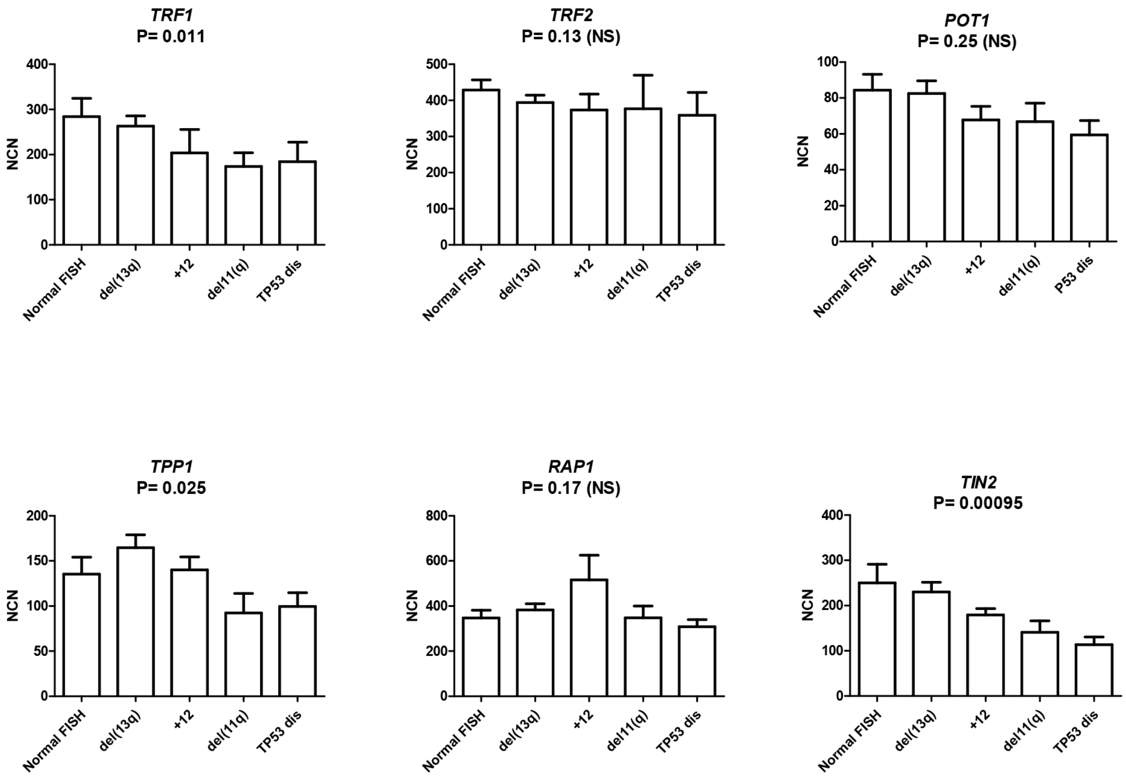

Supplementary Figure S2: *TP53*-disrupted showed the lowest levels of shelterin gene expression, which was significant for *TRF1* and *TPP1* and highly significant for *TIN2* (H test). NCN= normalized copy-number of mRNA.

Supplementary Table S1: Patients' characteristics

	**Median [range] or No. (%)**
Total number of patients	115
Median age, years [range]	69 [39-90]
Gender: Male/Female	77/38
Binet stages, *n* (%)	
A	56 (49)
B	24 (21)
C	35 (30)
Lymphocyte doubling time	
<6 months	49 (43)
≥6 months	66 (57)
CD38 expression	
negative (<7%)	87 (76)
positive (≥7%)	28 (24)
Cytogenetics	
del(17p)	14 (12)
del(11q)	15 (13)
trisomy 12	10 (9)
normal karyotype	18 (16)
del(13q) only abnormality	50 (43)
various abnormalities	8 (7)
complex karyotype	13 (11)
IGHV status	
mutated	65 (56)
unmutated	48 (42)
not determined	2 (2)

Supplementary Table S2: Characteristics of *TP53* mutations found in this CLL series and including the data from previously reported cancer cases from the IARC TP53 Database (http://www-p53.iarc.fr/)

Patient	Mutation (estimated % of mutated clone)	Position	Amino-acid change	Type of mutation	Impact	Cases reported by IARC (most common sites)
CLL #1	c.529_546del18 (50%)	Exon 5	p.177_182del	deletion of 6 amino acids without frameshift	(no data)	13 (anus, testicle)
CLL #2	c.701A>G (30%)	Exon 7	p.Y234C	missense	deleterious	144 (tonsil)
CLL #3	c.814G>T (70%)	Exon 7	p.V272L	missense	deleterious	42 (gingiva, tongue)
CLL #4	c.814G>T (50%)	Exon 8	p.V272L	missense	deleterious	42 (gingiva, tongue)
CLL #5	c.840 A>T (60%)	Exon 8	p.R280S	missense	deleterious	23 (hypopharynx, urinary tract)
CLL #6	c.536A>G (10%)	Exon 5	p.H179R	missense	deleterious	171 (ureter and recto-sigmoidal junction)
CLL #7	c.743G>A (50%)	Exon 7	p.R248Q	missense	deleterious	939 (female genital tract)
CLL #8	c.476C>T (50%)	Exon 5	p.A159V	missense	deleterious	55 (uterus, respiratory system)
CLL #9	c.542G>C (30%)	Exon 5	p.R181P	missense	deleterious	24 (sinus, bone)
CLL #10	c.523C>G (50%)	Exon 5	p.R175G	missense	deleterious	24 (hematopoietic tissue, lung)
CLL #11	c.818G>A (50%)	Exon 8	p.R273H	missense	deleterious	851 (endocrine, penis)
CLL #12	c.701A>G (30%)	Exon 7	p.Y234C	missense	deleterious	144 (tonsil)
CLL #13	c.733G>T (50%)	Exon 7	p.G245C	missense	deleterious	91 (peritoneum)
CLL #14	c.467G>C (100%)	Exon 5	p.R156P	missense	deleterious	45 (bone)
CLL #15	c.524G>A (20%)	Exon 5	p.R175H	missense	deleterious	1211 (urinary tract, head & neck)
	c.763A>T (20%)	Exon 7	p.I255F	missense	deleterious	42 (palate, peritoneum)
CLL #16	c.625_626del2 (70%)	Exon 6	p.R209Kfs6	frameshift	(no data)	44 (salivary glands)
CLL #17	c.618_634del (100%)	Exon 6	p.L206Ffs2X	frameshift	unknown	unknown
CLL #18	c.493C>T (60%)	Exon 5	p.Q165X	nonsense	deleterious	48 (nasal??, bladder)
	c.1025_1060del (40%)	Exon 10	p.R342Qfs39X	frameshift	unknown	unknown
CLL #19	c.584T>C (30%)	Exon 6	p.I195T	missense	deleterious	106 (uterus, palate)
	c.673-2A>C (15%)	Intron 7	-	splice site	(no data)	4 (hematopoietic tissue)
	c.842A>T (25%)	Exon 8	p.D281V	missense	deleterious	5 (bone, lymph node)
CLL #20	c.993+1G>A (80%)	Intron 9	-	splice site	(no data)	17 (pancreas, ovary)
